# Supplementary material for: Iron control of erythroid microtubule cytoskeleton as a potential target in treatment of iron-restricted anemia
Source: Nat Commun. 2021 Mar 12;12:1645. doi: 10.1038/s41467-021-21938-2 (PMC7955080; doi:10.1038/s41467-021-21938-2)
Supplement: Supplementary file 1 — Supplementary Information [file 41467_2021_21938_MOESM1_ESM.pdf]

Supplementary Materials: Figures

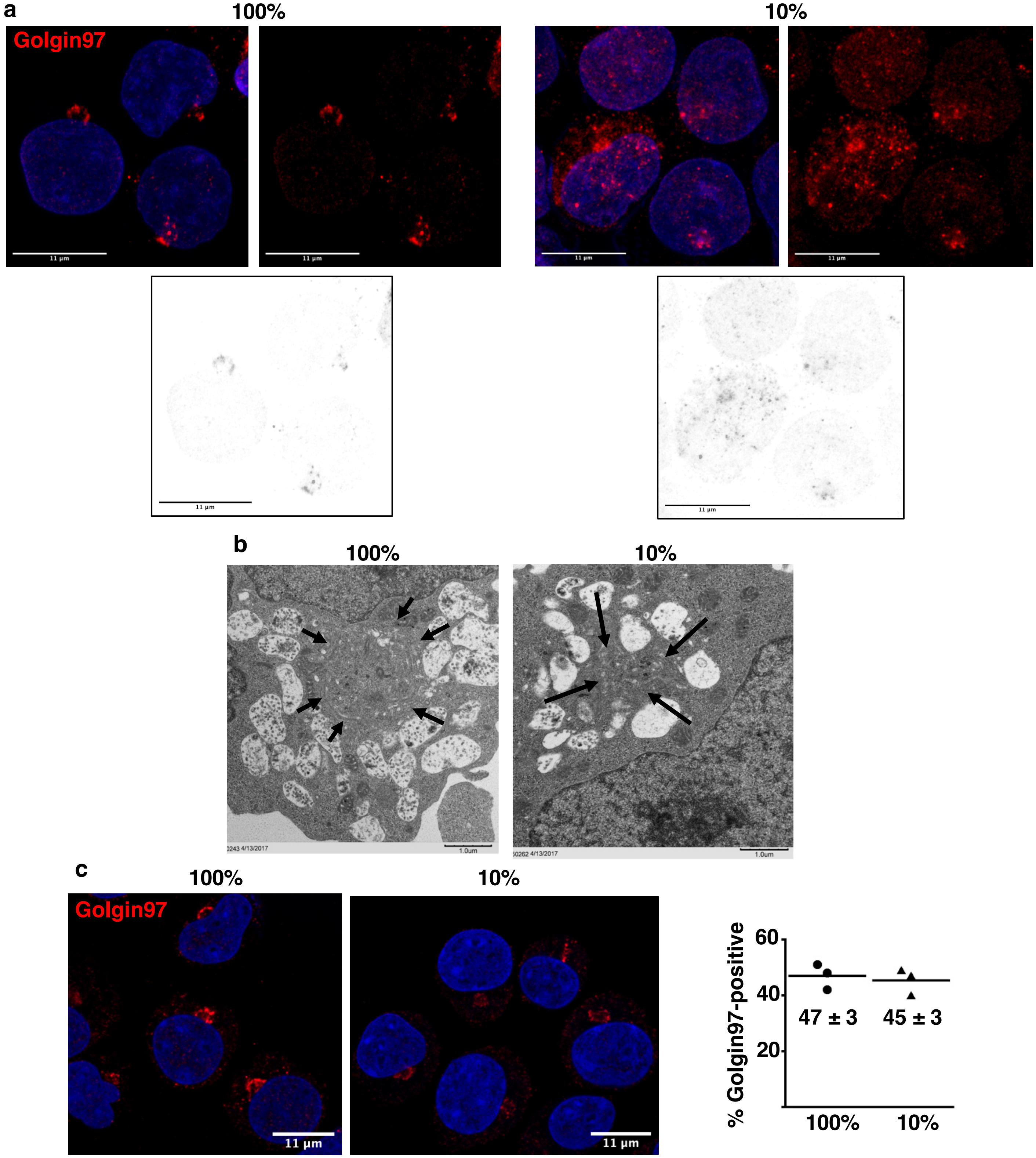

Supplementary Fig. 1. Erythroid Golgi perturbation with iron restriction. a, Disrupted Golgi in iron restricted erythroid progenitors. Immunofluorescence on human CD34+ progenitors cultured 3 days in erythroid medium with the indicated transferrin saturations. Red: Golgin97; blue: DAPI. Imaging conducted with Zeiss Airyscan superresolution microscopy; bottom: inverted grayscale. Representative results from two independent experiments. **b,** assessment of erythroid Golgi ultrastructure by transmission electron microscopy (TEM) on CD34+ cells cultured 3 days in erythroid media -/+ iron restriction. Arrows indicate organelle outer boundaries. Representative cells shown from two independent experiments. **c,** Retained Golgi in iron restricted granulocytic progenitors. Immunofluorescence on human CD34+ progenitors cultured 3 days in granulocytic medium with the indicated transferrin saturations. Red: Golgin97; blue: DAPI. Graph: % cells with intact Golgi, mean ± SEM; *n* = 3 biologically independent experiments.

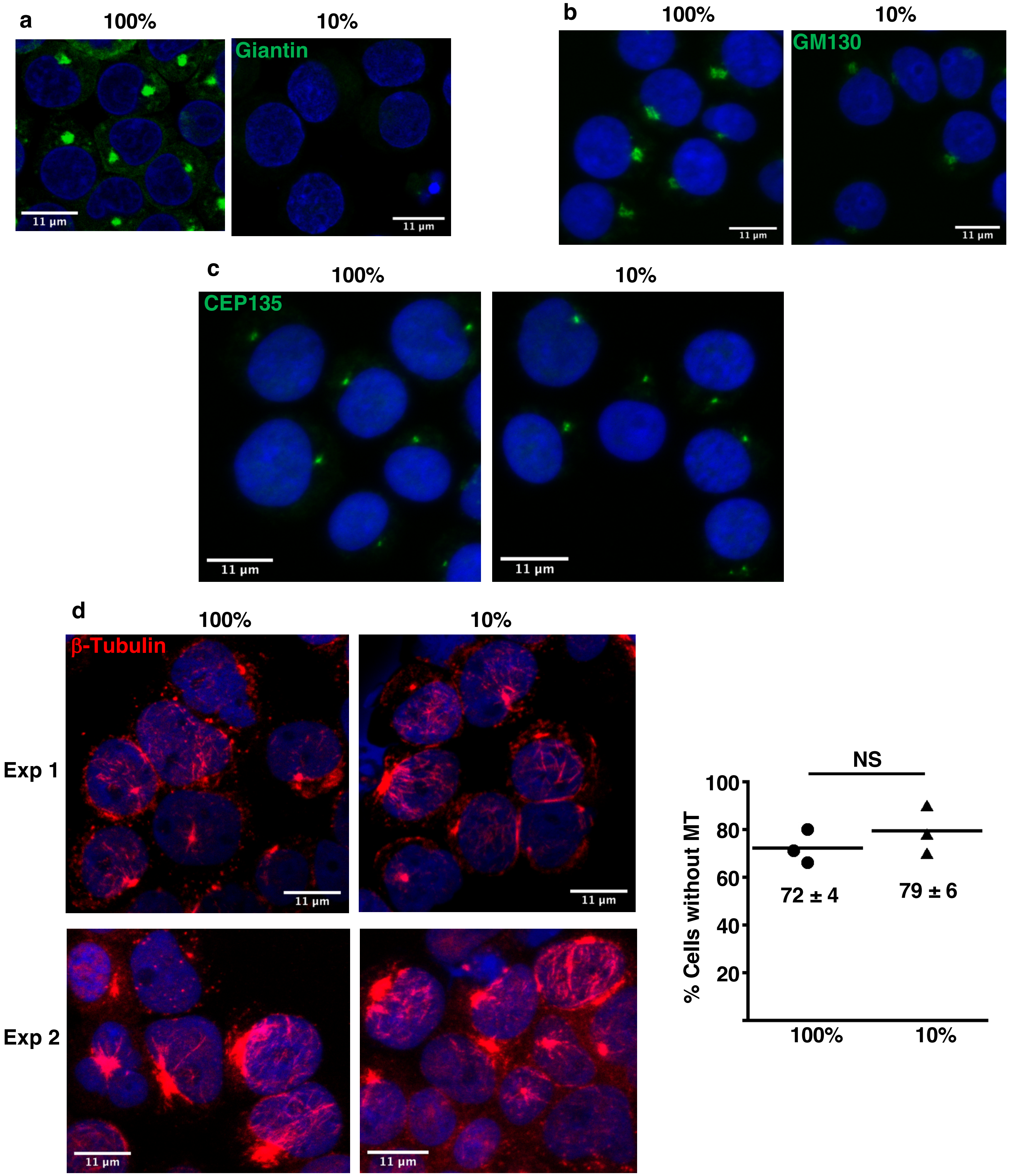

Supplementary Fig. 2. Assessment of additional Golgi markers; no effects of iron restriction on erythroid centrioles or on granulocytic microtubules. a, b, assessment of Golgi using immunofluorescence for additional markers on CD34+ cells cultured 3 days in erythroid media -/+ iron restriction. Green: Giantin (a) or GM130 (b); blue: DAPI. Representative results from 3 independent experiments. **c,** imaging of erythroid centrioles using immunofluorescence on CD34+ cells cultured 3 days in erythroid media -/+ iron restriction. Green: CEP135; blue: DAPI. Representative results from 3 independent experiments. **d,** granulocytic microtubule comparisons by immunofluorescence on human CD34+ progenitors cultured 3 days in granulocytic medium -/+ iron restriction. Red: β-Tubulin; blue: DAPI. Graph: % cells lacking microtubules (MT), mean ± SEM; *n* = 3 biologically independent experiments. NS: not significant, unpaired two-sided Student's *t* test.

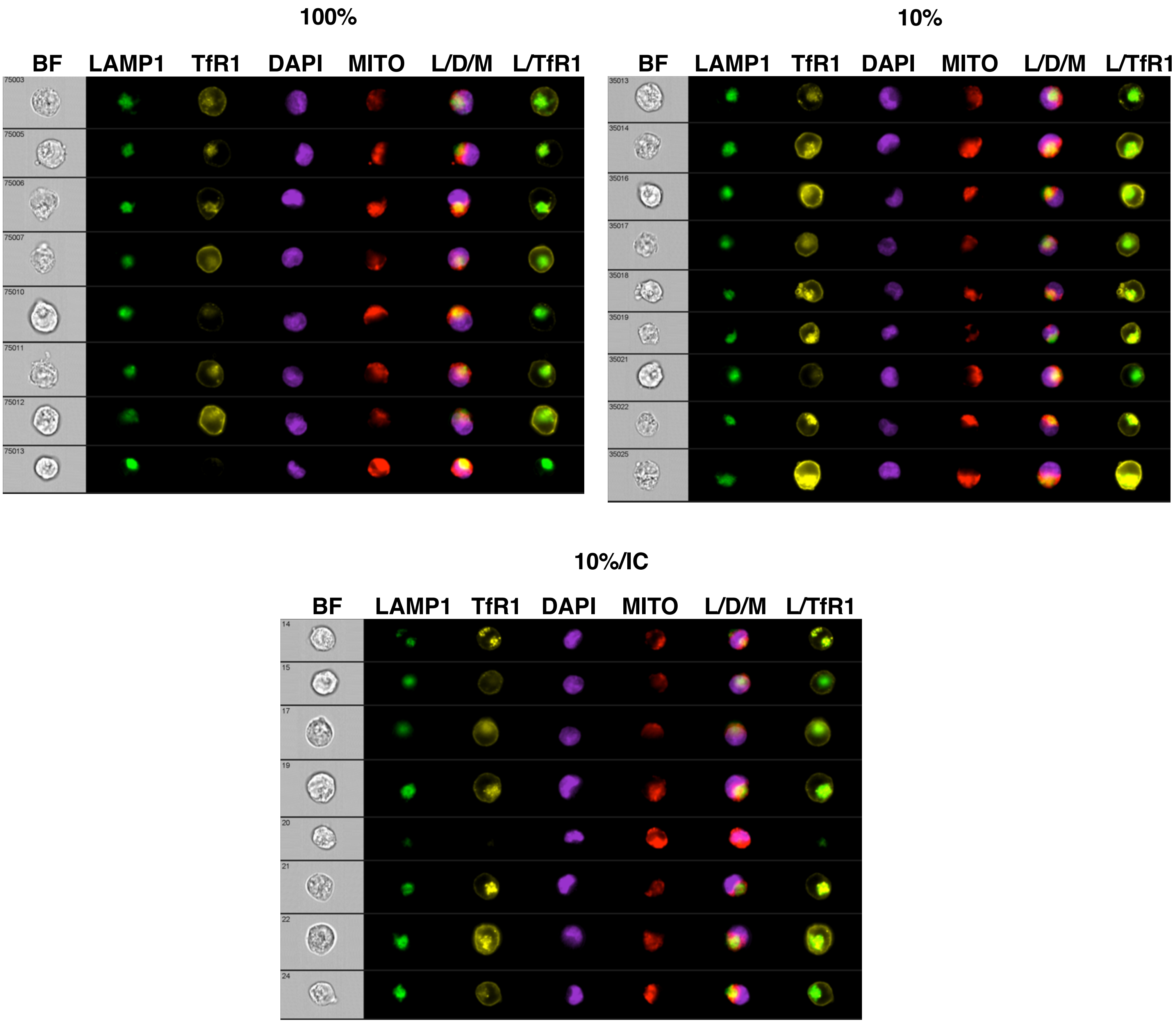

Supplementary Fig. 3. Imaging of transferrin receptor expression and localization in erythroid progenitors subjected to iron restriction and isocitrate treatment. visualization of erythroid transferrin receptor (TfR1 aka CD71, yellow) in relation to lysosome (LAMP1, green), nucleus (DAPI, blue), and mitochondria (MITO, red). CD34+ cells cultured 4 days in erythroid media -/+ iron restriction (10%) and isocitrate (IC) underwent immuno-staining for surface and intracellular TfR1 and LAMP1. Cells were co-stained with DAPI for nuclei and MitoTracker Red for mitochondria. Bright field (BF) and fluorescent images were obtained on an Amnis ImageStream flow cytometer with gating on viable, singlet, in-focus cells. Overlays of LAMP1 with nuclei/mitochondria and with TfR1 are shown in the last 2 columns.

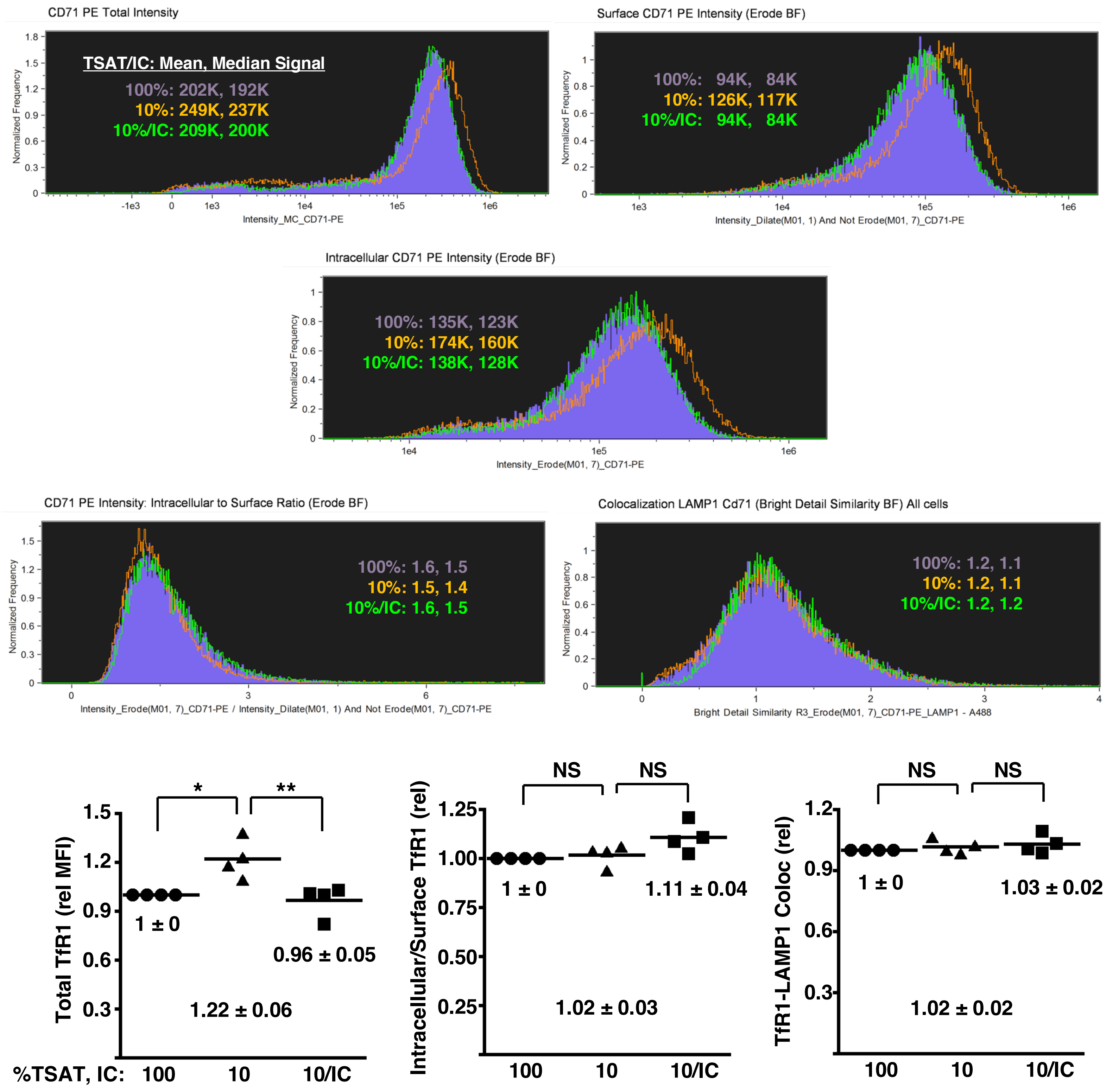

Supplementary Fig. 4. Quantitation of transferrin receptor expression and localization in erythroid progenitors subjected to iron restriction and isocitrate treatment. histogram plots of TfR1 levels (total, surface, intracellular) and distribution (intracellular/surface ratio, co-localization with lysosome), with mean and median signal intensity. Cells were cultured, stained and analyzed as in Supplementary Fig. 3, with each analysis using data acquired from >19,000 cells. Graphs depict relative mean values from 4 independent experiments ± SEM; *, **P = 0.017, 0.008, one-way ANOVA with Tukey post hoc test. Abbreviations: IC (isocitrate), BF (bright field), rel (relative), MFI (mean fluorescence intensity), Coloc (colocalization), NS (not significant).

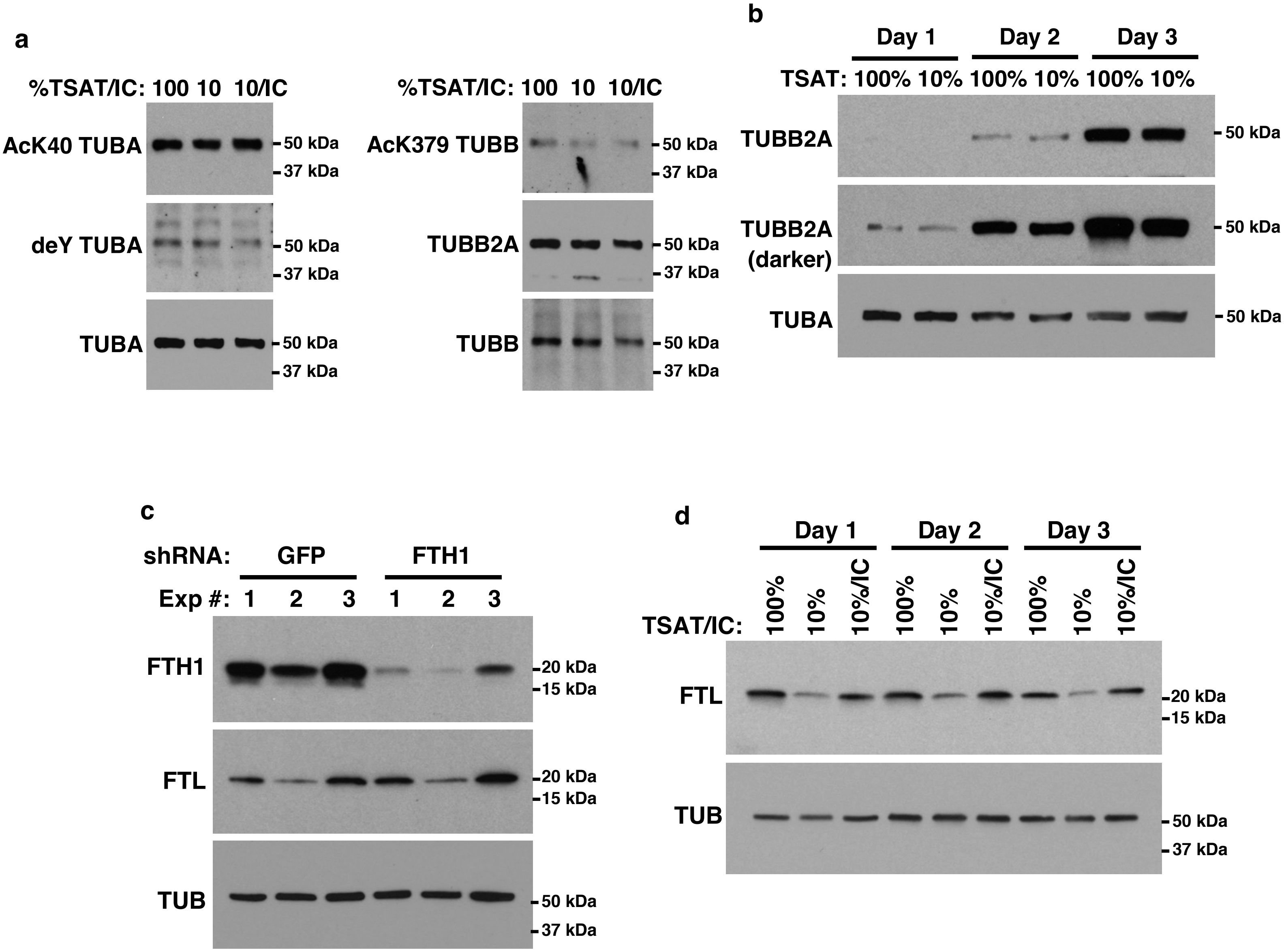

Supplementary Fig. 5. Effects of erythroid iron restriction on tubulin modifications, tubulin isoforms and ferritin light chain. a, no detectable effects of iron restriction or isocitrate on tubulin modifications (lysine acetylation, AcK or detyrosination, deY) or isoform levels (α, TUBA; β, TUBB; β-2A, TUBB2A). Immunoblot analysis on whole cell lysates from human CD34+ progenitors cultured 3 days in iron replete (TSAT 100%) or deficient (TSAT 10%) erythroid medium -/+ isocitrate (IC). Representative results from 3 independent experiments. **b,** the tissue-specific tubulin β-2A chain undergoes robust induction during erythroid differentiation but is unaffected by iron restriction. Immunoblot analysis on whole cell lysates from human CD34+ progenitors cultured 1-3 days in iron replete (TSAT 100%) or deficient (TSAT 10%) erythroid medium. Representative results from 3 independent experiments. **c,** specificity of antibody to ferritin heavy chain (FTH1). Immunoblot analysis of human CD34+ progenitors transduced with lentiviral shRNA constructs and cultured 3 days in iron replete erythroid medium. Results are shown from 3 independent experiments. **d,** ferritin light chain (FTL) responds to iron restriction and isocitrate in a manner similar to FTH1. Immunoblot analysis of human CD34+ progenitors cultured 1-3 days in iron replete or deficient erythroid medium -/+ isocitrate rescue (IC). Representative results from 3 independent experiments.

Erythroid Day 2

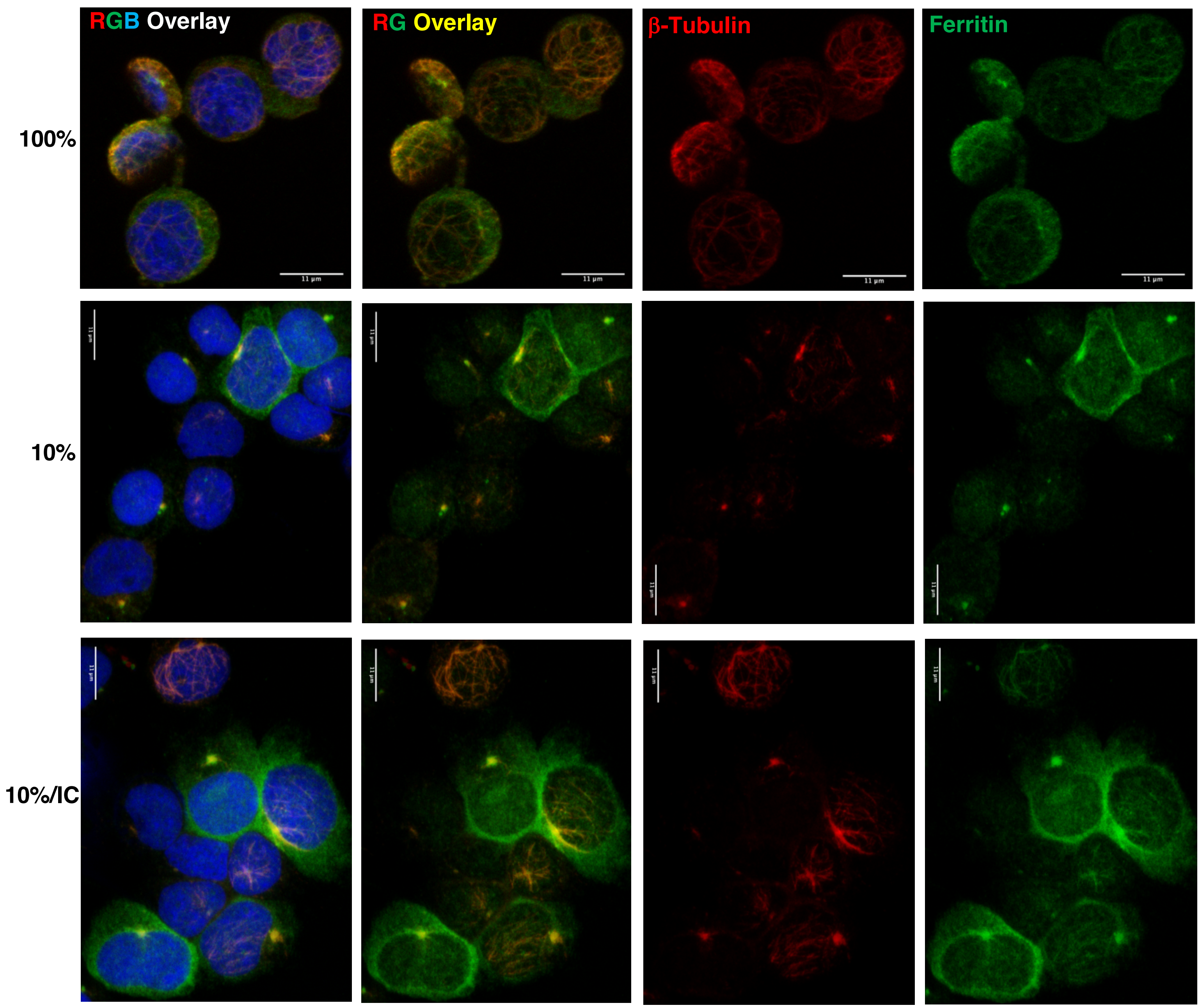

Supplementary Fig. 6. Partial co-localization of ferritin with microtubules. imaging of erythroid microtubules and ferritin using immunofluorescence on CD34+ cells cultured 2 days in erythroid media -/+ iron restriction (10%) and isocitrate (IC). Representative results from 3 independent experiments. Green: ferritin; red: β-Tubulin; blue: DAPI. Representative results from 3 independent experiments.

Erythroid Day 3

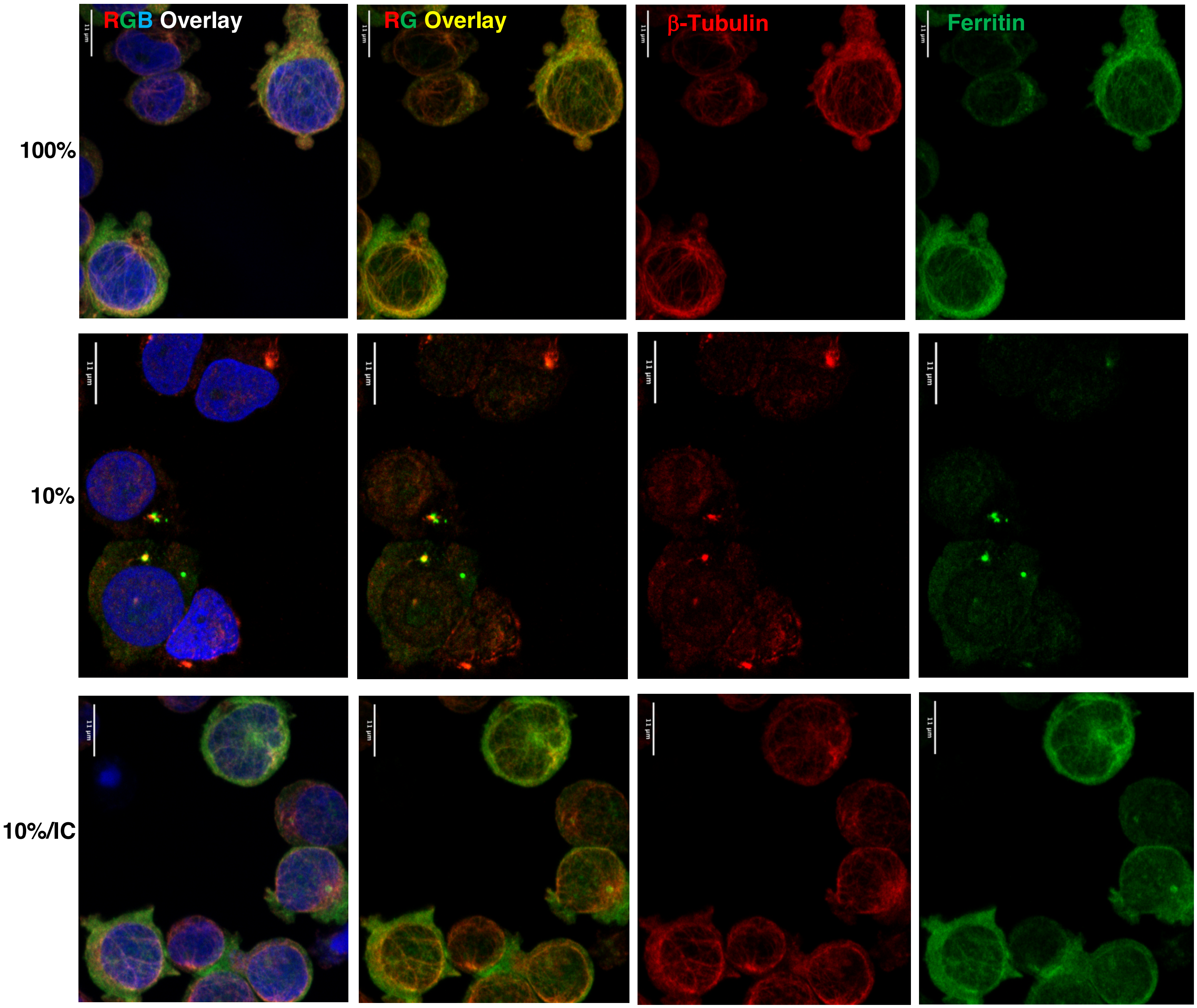

Supplementary Fig. 7. Partial co-localization of ferritin with microtubules. imaging of erythroid microtubules and ferritin using immunofluorescence on CD34+ cells cultured 3 days in erythroid media -/+ iron restriction (10%) and isocitrate (IC). Representative results from 3 independent experiments. Green: ferritin; red: β-Tubulin; blue: DAPI. Representative results from 3 independent experiments.

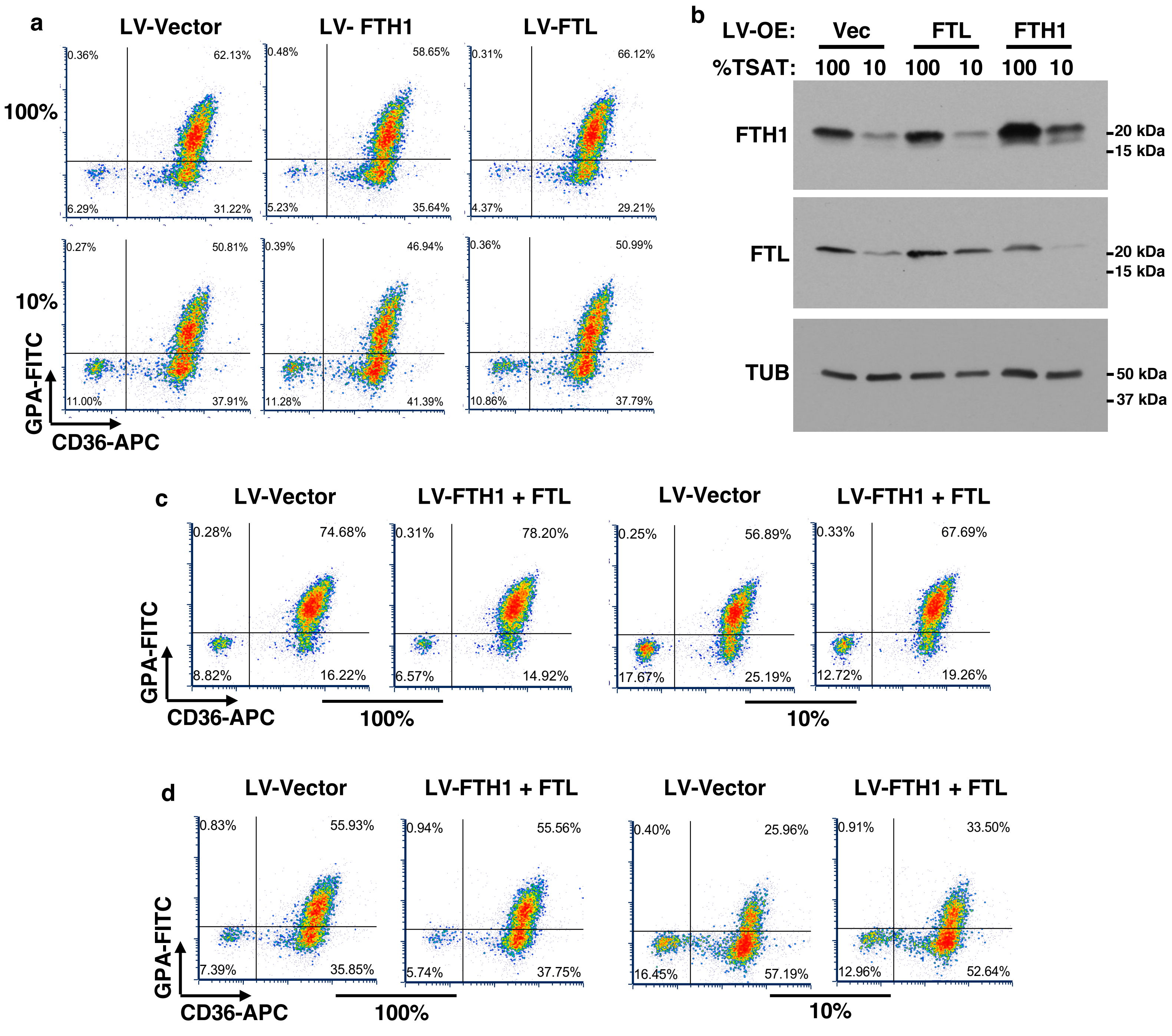

Supplementary Fig. 8. Requirement of coupled ferritin chain enforcement for reversal of erythroid iron restriction response. a, lentiviral (LV) enforcement of individual ferritin heavy (FTH1) or light (FTL) chain in human CD34+ progenitors subjected to 4 days culture in iron replete (100% TSAT) or deficient (10% TSAT) erythroid medium, followed by flow cytometry for differentiation. **b,** ferritin chain expression by immunoblot on whole cell lysates of cells transduced for lentiviral overexpression (LV-OE) and cultured as in **a**. Representative results from 3 independent experiments. **c, d,** independent biological replicates of the flow cytometry study in **Fig. 4E**, showing reversal of iron restriction response with coexpression of FTH1 and FTL.

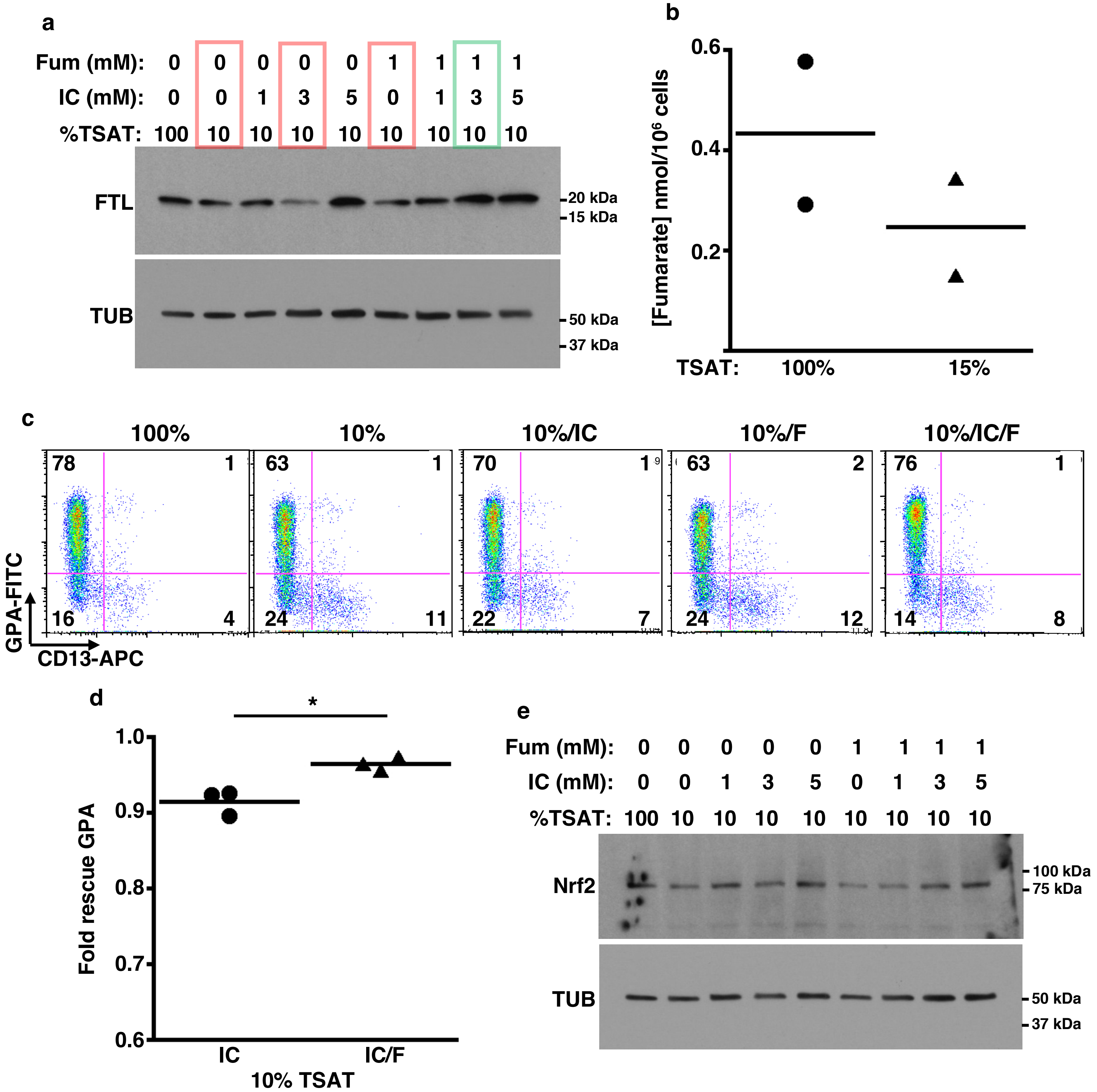

Supplementary Fig. 9. Cooperation of isocitrate and fumarate in counteracting erythroid iron restriction. a, ferritin light chain (FTL) levels by immunoblot on whole cell lysates from human CD34+ progenitors cultured 2 days in iron-replete (100% TSAT) or deficient (10% TSAT) erythroid medium -/+ 1-5 mM isocitrate (IC) and 1 mM fumarate (Fum). Colored boxes highlight synergy of 3 mM IC with 1 mM Fum. Red: no or single metabolite treatment; green: combined metabolite treatment. Representative results from 3 independent experiments. **b,** Fumarate levels by NMR profiling of extracts from human CD34+ progenitors cultured 4 days in erythroid medium with indicated TSATs, mean ± SEM; n = 2 independent repeats. **c, d,** assessment of erythoid differentiation by flow cytometry on human CD34+ progenitors cultured 5 days in iron-replete (100% TSAT) or deficient (10% TSAT) erythroid medium -/+ 5 mM isocitrate (IC) and 1 mM fumarate (F). GPA: glycophorin A. Graph: mean %GPA+ cells relative to value with 100% TSAT. Error bars, SEM; n = 3 biologically independent experiments; $^*P$ = 0.019, unpaired two-sided Student's t test. **e,** Nrf2 levels by immunoblot on whole cell lysates from human CD34+ progenitors cultured as in **a**. Representative results from 3 independent experiments.

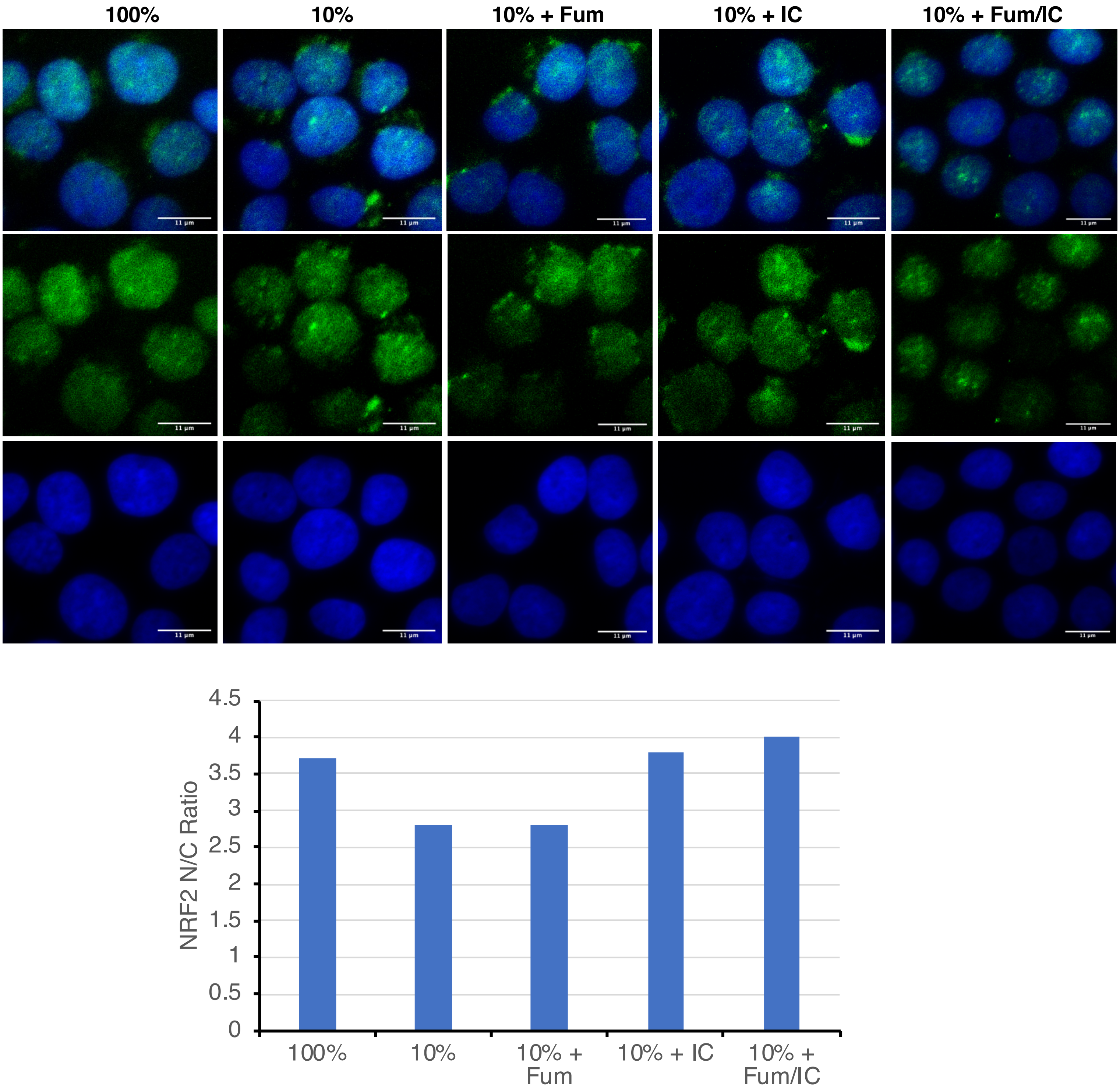

Supplementary Fig. 10. Assessment of Nrf2 nuclear localization in response to iron restriction, isocitrate, and fumarate. Nrf2 localization demonstrated by immunofluorescence on human CD34+ progenitors cultured 2 days in iron-replete (100% TSAT) or deficient (10% TSAT) erythroid medium -/+ 3 mM isocitrate (IC) and 1 mM fumarate (F). Green: Nrf2; blue: DAPI. Representative fields are shown from one experiment. Graph: nuclear/cytoplasmic ratio of Nrf2 (33-54 cells per condition).

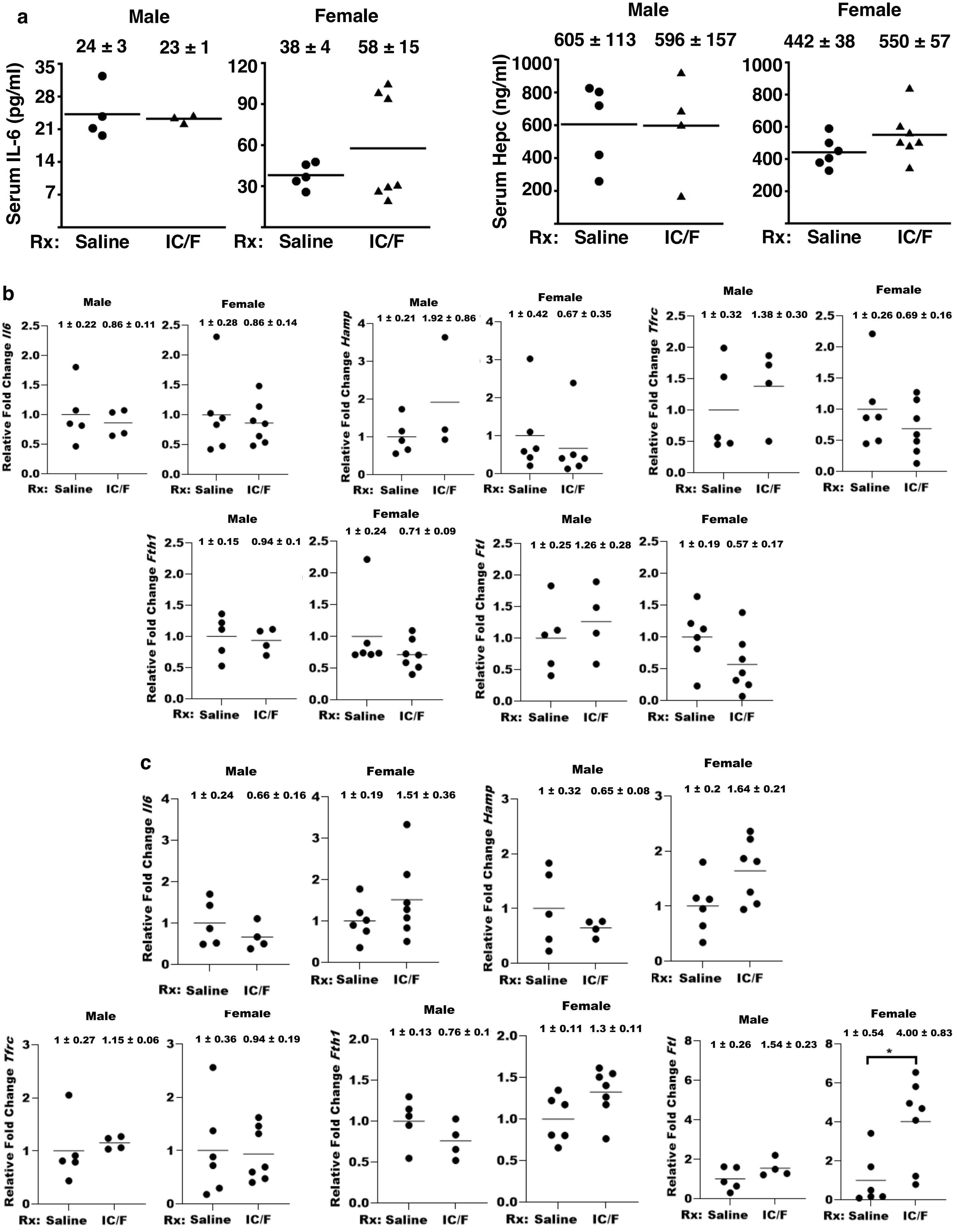

a
Male
24 ± 3
23 ± 1
Female
38 ± 4
58 ± 15
Serum IL-6 (pg/ml)
Rx: Saline IC/F
Male
605 ± 113
596 ± 157
Female
442 ± 38
550 ± 57
Serum Hepc (ng/ml)
Rx: Saline IC/F
b
Male
1 ± 0.22 0.86 ± 0.11
Female
1 ± 0.28 0.86 ± 0.14
Relative Fold Change Il6
Male
1 ± 0.21 1.92 ± 0.86
Female
1 ± 0.42 0.67 ± 0.35
Relative Fold Change Hamp
Male
1 ± 0.32 1.38 ± 0.30
Female
1 ± 0.26 0.69 ± 0.16
Relative Fold Change Tfrc
Male
1 ± 0.15 0.94 ± 0.1
Female
1 ± 0.24 0.71 ± 0.09
Relative Fold Change Fth1
Male
1 ± 0.25 1.26 ± 0.28
Female
1 ± 0.19 0.57 ± 0.17
Relative Fold Change Ftl
Rx: Saline IC/F
c
Male
1 ± 0.24 0.66 ± 0.16
Female
1 ± 0.19 1.51 ± 0.36
Relative Fold Change Il6
Male
1 ± 0.32 0.65 ± 0.08
Female
1 ± 0.2 1.64 ± 0.21
Relative Fold Change Hamp
Male
1 ± 0.27 1.15 ± 0.06
Female
1 ± 0.36 0.94 ± 0.19
Relative Fold Change Tfrc
Male
1 ± 0.13 0.76 ± 0.1
Female
1 ± 0.11 1.3 ± 0.11
Relative Fold Change Fth1
Male
1 ± 0.26 1.54 ± 0.23
Female
1 ± 0.54 4.00 ± 0.83
*
Relative Fold Change Ftl
Rx: Saline IC/F

Supplementary Fig. 11. Minimal effect of oral isocitrate-fumarate treatment (IC/F) on iron-related parameters. a, mice as in Figure 7b, with ACDI -/+ treatment were analyzed for serum IL-6 and hepcidin (Hepc) levels. Grubb's test eliminated outliers. Numbers reflect mean ± SEM. Unpaired 2-sided Student's *t* test showed no differences. *n* (IL-6) = 4 males and 5 females for saline; 3 males and 7 females for IC/F. *n* (Hepc) = 5 males and 6 females for saline; 4 males and 7 females for IC/F. **b, c** spleen and liver transcripts, respectively, by qRT-PCR, on same cohorts as in **a**. Statistical analysis was conducted as in **a**. *n* = 5 males and 6 females for saline; 4 males and 7 females for IC/F. *P = 0.012, unpaired two-sided Student's *t* test.

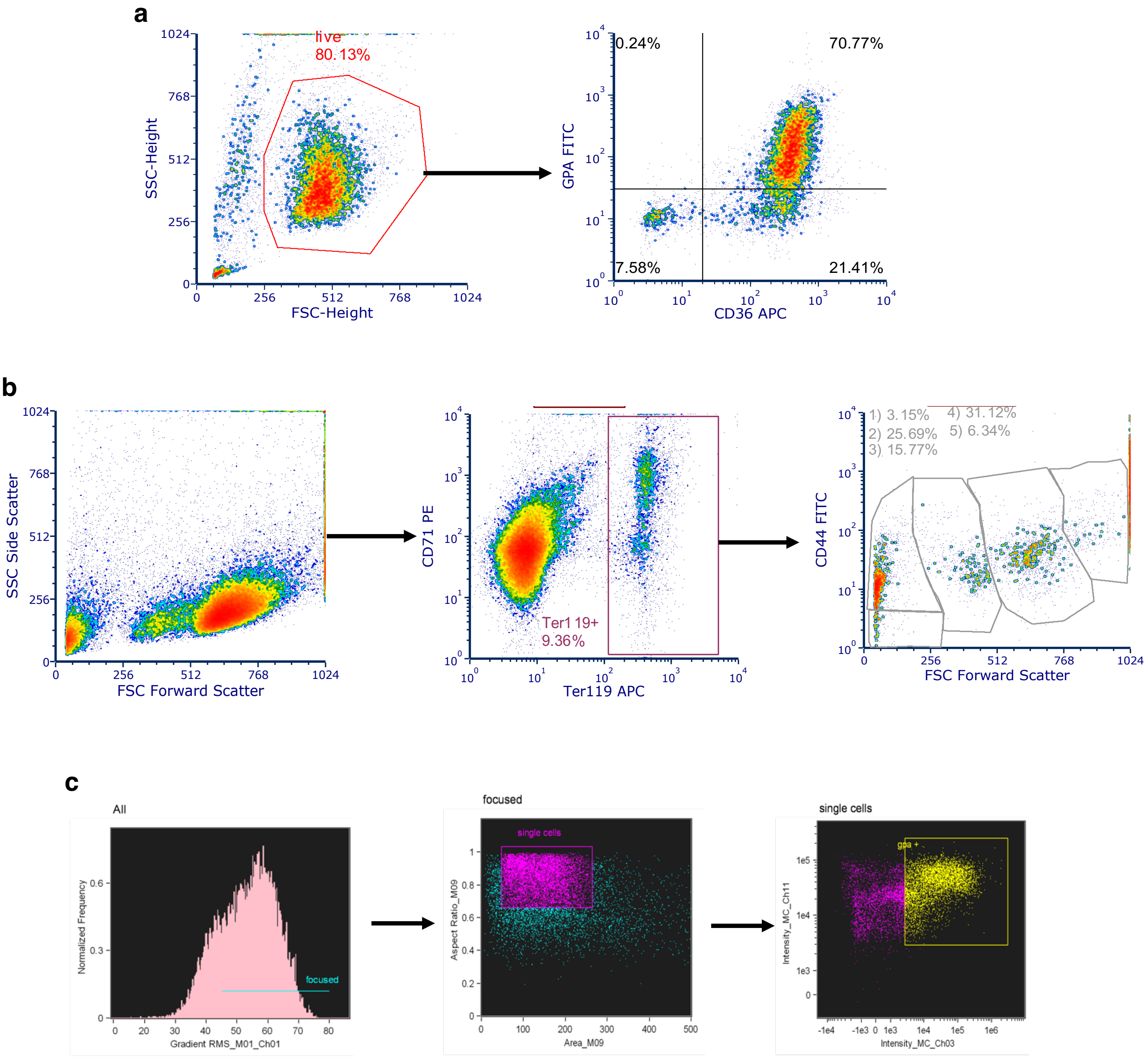

Supplementary Fig. 12. Gating Strategies. a, Gating strategy for human progenitor analysis in Figure 4 (c, e), Supplementary Figure 8 (a, c, d), and Supplementary Figure 9c. **b,** Gating strategy for murine splenic erythroid progenitor analysis in Figure 7c. **c,** Gating strategy for Amnis ImageStream analysis of human progenitors in Supplementary Figures 3-4.

Supplementary Tables

Supplementary Table 1. Protein levels by lineage in human marrow progenitors.

	Lymphoid	Monocytic	Granulocytic	Erythroid
**STMN1**	10.484	47.546	35.227	62.346 ↑
**FTH1**	0.511	2.634	0.174	20.517 ↑
**MAPRE1**	13.079	11.382	10.586	5.437 ↓
**MAPRE2**	2.226	1.839	1.734	0.761 ↓
**RMDN1**	0.442	0.765	1.187	0.323 ↓
**MAP4**	2.202	1.227	0.209	0.190 ↓

Proteomic data mined from Hennrich et al[1]. Quantitation consists of normalized label-free sum calculated from 16-49 donor samples per lineage, using a normalization formula described by the authors.

Supplementary Table 2. List of primers used in qPCR.

Gene	Alignment	Sequence	NCB1 ID
Tfrc	Forward	TAGAGGCGCTTCCTAGTACTCC	NM_011638.4
	Reverse	GTCTCCTCCGTTTCAGCCAG	
Ftl	Forward	ATGACCTCTCAGATTCGTCAG	NM_010240.2
	Reverse	CTAGTCGTGCTTGAGAGTGAG	
Fth1	Forward	CTCATGAGGAGAGGGAGCAT	NM_010239.2
	Reverse	GTGCACACTCCATTGCATTC	
Hamp	Forward	CCTATCTCCATCAACAGATG	NM_032541.2
	Reverse	AACAGATACCACACTGGGAA	
Il6	Forward	ACAACGATGATGCACTTGCAG	In house
	Reverse	GCATTGGAAATTCGGGTAGGAA	
B2m	Forward	attcacccccactgagactg	In house
	Reverse	tgctatttctttctgcgtgc	

Supplementary Methods

Electron microscopy. Transmission electron microscopy on cultured human progenitors was carried out essentially as described[2]. Briefly, the harvested cells were washed in PBS and fixed in 2% paraformaldehyde plus 2% glutaraldehyde in PBS for 20 minutes at room temperature. Samples were further fixed in 1% osmium tetroxide for 30 minutes at room temperature, followed by embedding and polymerization at 65°C for 24 hours. 70 nM sections arrayed on 200 mesh grids were stained with uranyl acetate and lead citrate. Grids underwent carbon coating to decrease conductance and were analyzed on a JEOL 1230 (JEOL, Peabody, MA) electron microscope at 80 kV. Images were taken with a Scientific Instruments of America 4K X 4K CCD camera.

Imaging flow cytometry. Amnis ImageStream® analysis of cultured human progenitors followed our published approach[2]. Specifically, harvested cells were resuspended in serum-free, phenol red-free IMDM and subjected to initial staining with 30 nM MitoTracker Deep Red FM® (M22426, ThermoFisher Scientific) for 15 minutes at 37°C. The cells were washed with 37°C PBS and fixed in 4% fresh paraformaldehyde for 15 minutes at room temperature with occasional mixing. PBS was then added to adjust the paraformaldehyde to 1% and cells were stored at 4°C overnight. The cells were then washed with PBS and permeabilized 15 minutes at room temperature with 0.08% Triton X-100, 1% FBS in PBS. For immunostaining, the cells underwent resuspension in 100 µl PBS with 2% FBS, followed by addition of 4 µl PE-anti-CD71 (ThermoFisher, 12-0719-42 [mouse monoclonal OKT9]) and 5.5 µl Alexa Fluor 488-anti-LAMP1 (ThermoFisher, MA5-18121 [mouse monoclonal H4A3]), with incubation at room temperature for 30 minutes

followed by washing twice with PBS. Immediately prior to analysis, cells were stained with 3 µg/ml DAPI in PBS with 0.01% Triton X-100 for 15-30 minutes at room temperature. The samples were then concentrated to 10^6 cells per 50 µl and run on an Amnis ImageStreamX Mark II cytometer (EMD Millipore, Billerca, MA). Data analysis was conducted using the IDEAS® software package (EMD Millipore) with gating on viable, singlet, in-focus cells (Supplementary Fig. 12c).

Cell fumarate quantitation. Metabolite quantitation occurred as described[3]. ~5×10^7 cells washed twice in ice-cold PBS were suspended in ice-cold distilled H_2O and then supplemented with an equal volume of cold (−20°C) absolute methanol. After vortexing briefly, samples were frozen in a dry ice bath for 30 minutes and then thawed in a wet ice bath for 10 minutes. After centrifugation (18,000 × g, 10 minutes), the supernatant was collected, and the pellet underwent repeat extraction with 50% v/v cold (−20°C) methanol, followed by pooling of first and second supernatants. These extracts were then evaporated to dryness, and re-dissolved in 150 µl of distilled H_2O. After briefly heating to 90°C, samples were cooled on ice, centrifuged (18,000 × g, 10 minutes), and then supernatants were harvested for desiccation by Speed-Vac.

For NMR spectroscopy, the extracts were dissolved in 300 µl D_2O with 1.5 mM (trimethylsilyl-2,2,3,3-tetradeuteropropioninc acid (TSP) and placed in 3 mm NMR tubes for high resolution ^{1}H NMR analysis. 1D ^{1}H NMR spectra were obtained by a 16.4T Varian INOVA (700MHz 1H, Varian Instruments) equipped with a 5 mm indirect cold probe. Free induction decays (FIDs) underwent acquisition using a one-pulse sequence with a total repetition time (TR) of 12.65s, number of transients (nt) of 64, and flip angle of 90°.

Spectral processing employed ACD/ 1D NMR Manager (version 12.0; Advanced Chemistry Development, Inc., Toronto, ON, Canada). FIDs were zero filled to 64,000 points, and an exponential line broadening of 0.1 Hz was employed, followed by Fourier transformation. Phase and baseline-corrected spectra were referenced to the TSP peak at 0.00 ppm. Metabolite identification and quantification employed Chenomx NMR Suite (version 5.1; Chenomx Inc., Edmonton, Canada).

Analysis of iron-related parameters in ACDI mice. Mice underwent ACDI induction and treatment as in Fig. 7b and c. Two days after the last oral gavage, mice were anesthetized with isoflurane and euthanized by cervical dislocation. Immediately after, blood was collected by heart puncture for serum studies and portal veins were perfused with 10 ml Hanks' Balanced Salt Solution (HBBS) containing 0.5 mM EGTA, followed by a 5 ml HBSS flush.

For serum studies, blood samples underwent clotting at room temperature for 30 minutes followed by a microfuge spin at 3000 g at room temperature for 15 minutes. Serum was collected and stored in aliquots at -20°C. For measurements, samples were thawed and diluted (1:2 for IL-6 and 1:10 for hepcidin) in buffers provided by the kit manufacturers. IL-6 was quantified using the IL-6 Mouse Uncoated ELISA Kit (Invitrogen, 88-7064). Hepcidin levels were measured with the Hepcidin Murine-Compete™ ELISA kit (Intrinsic Lifesciences, HMC-001). Light absorbance signals were measured with a SpectraMax Plus 384 microplate reader (Molecular Devices).

For transcript measurements, livers and spleens underwent homogenization of 50 mg tissue in 1 ml TRIzol (Invitrogen,15596026) using a TissueLyser II Homogenizer

(Qiagen). After chloroform extraction, RNA underwent precipitation with 70% ethanol, redissolving, purification using the RNeasy kit (Qiagen, 74106), and quantification with a NanoDrop 2000 (Thermo Scientific). cDNA was synthesized from 1000 µg total RNA with an iScript cDNA synthesis kit (Biorad, 1708891) according to manufacturer's instructions. For quantitative real-time PCR, cDNA, forward and reverse primers, and 2x SensiMix SYBR & Fluorescein Kit (Bioline, QT615-05) master mix were combined and analyzed on the CFX Connect Real-Time System (Biorad). β2-microglobulin (*B2m*) was used as an internal reference gene. Primers pairs (designed using NCBI-Primer Blast http://www.ncbi.nlm.nih.gov/tools/primer-blast or in house for Leitinger Lab) are shown above in Supplementary Table 2. Amplification efficiency was determined for each primer pair using a dilution series of sample cDNA. Relative quantification of gene expression was derived using the double delta CT formula.

Supplementary References

1. Hennrich, M.L., *et al.* Cell-specific proteome analyses of human bone marrow reveal molecular features of age-dependent functional decline. *Nat. Commun.* **9**, 4004 (4001-4018) (2018).
2. Khalil, S., *et al.* A specialized pathway for erythroid iron delivery through lysosomal trafficking of transferrin receptor 2. *Blood Adv.* **1**, 1181-1194 (2017).
3. Bullock, G.C., *et al.* Iron control of erythroid development by a novel aconitase-associated regulatory pathway. *Blood* **116**, 97-108 (2010).
