# Supplementary material for: Iron control of erythroid microtubule cytoskeleton as a potential target in treatment of iron-restricted anemia
Source: Nat Commun. 2021 Mar 12;12:1645. doi: 10.1038/s41467-021-21938-2 (PMC7955080; doi:10.1038/s41467-021-21938-2)
Supplement: Supplementary file 3 — Reporting Summary [file 41467_2021_21938_MOESM3_ESM.pdf]

nature research

Corresponding author(s): Adam Goldfarb

Last updated by author(s): Jan 15, 2021

Reporting Summary

Nature Research wishes to improve the reproducibility of the work that we publish. This form provides structure for consistency and transparency in reporting. For further information on Nature Research policies, see Authors & Referees and the Editorial Policy Checklist.

Statistics

For all statistical analyses, confirm that the following items are present in the figure legend, table legend, main text, or Methods section.

n/a	Confirmed	
☐	☒	The exact sample size (*n*) for each experimental group/condition, given as a discrete number and unit of measurement
☐	☒	A statement on whether measurements were taken from distinct samples or whether the same sample was measured repeatedly
☐	☒	The statistical test(s) used AND whether they are one- or two-sided<br>*Only common tests should be described solely by name; describe more complex techniques in the Methods section.*
☒	☐	A description of all covariates tested
☐	☒	A description of any assumptions or corrections, such as tests of normality and adjustment for multiple comparisons
☐	☒	A full description of the statistical parameters including central tendency (e.g. means) or other basic estimates (e.g. regression coefficient) AND variation (e.g. standard deviation) or associated estimates of uncertainty (e.g. confidence intervals)
☐	☒	For null hypothesis testing, the test statistic (e.g. *F*, *t*, *r*) with confidence intervals, effect sizes, degrees of freedom and *P* value noted<br>*Give P values as exact values whenever suitable.*
☒	☐	For Bayesian analysis, information on the choice of priors and Markov chain Monte Carlo settings
☒	☐	For hierarchical and complex designs, identification of the appropriate level for tests and full reporting of outcomes
☒	☐	Estimates of effect sizes (e.g. Cohen's *d*, Pearson's *r*), indicating how they were calculated

Our web collection on statistics for biologists contains articles on many of the points above.

Software and code

Policy information about availability of computer code

Data collection	Flow cytometry: CellQuest Pro; Fluorescence microscopy: Zen; Imaging cytometry (Amnis): INSPIRE
Data analysis	Flow cytomtery: FCS Express 6 Flow (De Novo Software); Fluorescence microscopy: Fiji ImageJ version 2.0.0 open source software (imagej.net/Fiji); Imaging cytometry (Amnis): IDEAS® 4.0 (EMD Millipore)

For manuscripts utilizing custom algorithms or software that are central to the research but not yet described in published literature, software must be made available to editors/reviewers. We strongly encourage code deposition in a community repository (e.g. GitHub). See the Nature Research guidelines for submitting code & software for further information.

Data

Policy information about availability of data

All manuscripts must include a data availability statement. This statement should provide the following information, where applicable:

- Accession codes, unique identifiers, or web links for publicly available datasets
- A list of figures that have associated raw data
- A description of any restrictions on data availability

All data relevant to this paper are available from the corresponding author. Figures with associated raw data include: Main Figures 1-7 and Supplemental Figures 1, 2, 4, 9, 11. There are no restrictions on data availability.

Field-specific reporting

Please select the one below that is the best fit for your research. If you are not sure, read the appropriate sections before making your selection.

☒ Life sciences ☐ Behavioural & social sciences ☐ Ecological, evolutionary & environmental sciences

For a reference copy of the document with all sections, see nature.com/documents/nr-reporting-summary-flat.pdf

Life sciences study design

All studies must disclose on these points even when the disclosure is negative.

Sample size	Sample sizes for both in vitro and animal experiments were determined by prior experience in our lab with the parameters studied. Specifically, our prior immunofluorescence experiments on human erythroid progenitors published in Blood Advances (Khalil et al., A specialized pathway for erythroid iron delivery through lysosomal trafficking of transferrin receptor 2. Blood Adv., 1(15):1181-94, 2017. PMCID: PMC5728310) indicated sufficiency of 3 independent repeats. Our prior experiments with flow cytometry and immunoblot on human erythroid progenitors (Khalil et al. Iron modulation of erythropoiesis is associated with Scribble-mediated control of the erythropoietin receptor. J. Exp. Med., 215(2):661-79, 2018. PMCID: PMC5789406.) supported the need for 3 independent repeats. Our 12-year experience with murine models of iron restricted anemia with isocitrate treatment (see Bullock et al. Iron control of erythroid development by a novel aconitase-associated regulatory pathway. Blood, 116:97-108, 2010. PMCID: PMC2904585) dictated the design of the current trial, indicating a typical requirement of 10 animals per group to determine treatment effects.
Data exclusions	For animal experiments, pre-established exclusion criteria consisted of defining outliers by performing a Grubb's test on all datasets. This was done to exclude animals with incidental health problems. The only outliers identified were in Extended Data Figure 11, as indicated in Figure legend.
Replication	Experiments were replicated independently three times, as indicated in legends. All attempts at replication were successful.
Randomization	For mouse anemia trials, mice were assigned to treatment or control group in manner to match the starting blood counts between the groups. For the vitro experiments, each sample was divided and subjected to all treatments, such that randomization was not needed.
Blinding	The identities of the marrow samples were masked prior to their evaluation. For the other experiments, blinding was not conducted because the methodology provided for rigorously objective quantification of the parameters to be analyzed.

Reporting for specific materials, systems and methods

We require information from authors about some types of materials, experimental systems and methods used in many studies. Here, indicate whether each material, system or method listed is relevant to your study. If you are not sure if a list item applies to your research, read the appropriate section before selecting a response.

Materials & experimental systems

n/a	Involved in the study	
☐	☒	Antibodies
☐	☒	Eukaryotic cell lines
☒	☐	Palaeontology
☐	☒	Animals and other organisms
☐	☒	Human research participants
☒	☐	Clinical data

Methods

n/a	Involved in the study	
☒	☐	ChIP-seq
☐	☒	Flow cytometry
☒	☐	MRI-based neuroimaging

Antibodies

Antibodies used	1. mouse monoclonal anti-Golgin97 (Invitrogen A-21270), 2. rabbit polyclonal anti-Giantin/GOLGB1 (Sigma HPA011008), 3. rabbit polyclonal anti-Pericentrin (Abcam ab84542), 4. rabbit monoclonal anti-GM130 (Cell Signaling D6B1), 5. mouse monoclonal anti-pan-β-tubulin (Sigma T4026), 6. Alexa Fluor® 488-conjugated goat F(ab')2 anti-rabbit IgG (H+L) (Cell Signaling Technology 4412), 7. Alexa Fluor® 546-conjugated goat anti-mouse IgG (H+L) (Invitrogen A-11030), 8. rabbit monoclonal anti-FTH1 (Cell Signaling Technology, 4393), 9. rabbit polyclonal anti-FTL (Abcam, ab69090), 10. mouse monoclonal anti-TUBB (Sigma, T4026), 11. Mouse monoclonal anti-TUBB2A (LSBio, LS-C413488), 12. rabbit polyclonal anti-AcK379 TUBB2A (LSBio, LS-C412264), 13. mouse monoclonal anti-TUBA (Sigma, T9026), 14. rabbit monoclonal anti-AcK40 TUBA (Abcam, ab179484), 15. rabbit polyclonal anti-TUBA, Detyrosinated (deY, Millipore, AB3201), 16-17. HRP-conjugated goat anti-rabbit and anti-mouse IgG (H+L) (Invitrogen/ThermoFisher, 31460 and 62-6520), 18-19. mouse monoclonal FITC-anti-CD235a (GPA) and fluorochrome-isotype matched control (ThermoFisher/eBioscienceTM, 11-9987-82 and 11-4732-81), 20-21. mouse monoclonal APC-anti-CD36 and fluorochrome-isotype matched control (ThermoFisher/eBioscienceTM, 561822 and 555585), 22. Rat monoclonal APC-anti-Ter119 (BD Biosciences, 557909), 23. Rat monoclonal PE-anti-CD71 (BD PharmingenTM 561937), and 24. Rat monoclonal FITC-anti-CD44 (BD PharmingenTM 553133), 25. rabbit polyclonal anti-CEP135 (Millipore ABE-1857), 26. rabbit monoclonal anti-Nrf2 (Cell Signaling Technology 12721).

Validation	Antibodies 1-5, 9 and 25 have been demonstrated in data provided by supplier to recognize cognate human antigens; antibodies 1 and 5 derive from mice; antibodies 2-4, 9, and 25 all derive from rabbits. All were validated in our lab for immunofluorescence on human cells by virtue of demonstrating the correct organelle distribution on confocal microscopy. Antibodies 8-15 and 26 were indicated by supplier to recognize human antigens. All were validated by immunoblot on whole cell lysates of human progenitors, yielding either solitary or major dominant bands of the correct molecular weight. The antibodies to FTH1 and FTL (8-9) were further validated on immunoblot by demonstrating their decline with targeted shRNA-mediated knockdowns. Antibodies 18-21 were indicated by supplier to be specific for human antigens. They were validated on flow cytometry showing erythroid-specific staining of human progenitors using isotype matched controls to define background. Antibodies 22-24 were indicated by supplier to be murine specific. All were validated by flow cytometry on murine marrow showing lineage specific co-staining patterns, using isotype-matched controls to define background.

Eukaryotic cell lines

Policy information about cell lines

Cell line source(s)	HEK293T were provided by ATCC
Authentication	None
Mycoplasma contamination	Not tested
Commonly misidentified lines (See ICLAC register)	No commonly misidentified cell lines were used in this study.

Animals and other organisms

Policy information about studies involving animals; ARRIVE guidelines recommended for reporting animal research

Laboratory animals	Mice: 1) KRNxG7 strain, male and female, 6-12 weeks of age. 2) C57BL/6 strain, male and female, 7-30 weeks of age. Mice were housed in specific pathogen-free conditions with 12-hour light/dark cycles at a temperature of 21 +/- 1.5C with humidity of 50 +/- 10%.
Wild animals	No wild animals were used in this study.
Field-collected samples	No field-collected samples were used in this study.
Ethics oversight	All animal studies were approved by the University of Virginia Institutional Animal Care and Use Committee (IACUC).

Note that full information on the approval of the study protocol must also be provided in the manuscript.

Human research participants

Policy information about studies involving human research participants

Population characteristics	To select marrow samples for immunostaining, one author (Dr. Gru) conducted a natural language search using the lab information system to identify pre-existing samples associated with the diagnosis of anemia of chronic disease. Pre-existing control samples were selected on the basis of normal marrow findings in the absence of anemia. To select blood samples for immunostaining, the clinical lab director Dr. Dede Haverstick used the lab information system to identify freshly discarded peripheral blood samples associated with the diagnosis of iron deficiency anemia, as well as age-matched non-anemic controls. These selection criteria yielded a population of male and female adults ranging in age from ~20-80 years old.
Recruitment	This study had no patient recruitment. Discarded samples were selected for study strictly based on diagnostic search criteria.
Ethics oversight	Approval was issued by the University of Virginia Institutional Review Board (HSR#13310)

Note that full information on the approval of the study protocol must also be provided in the manuscript.

Flow Cytometry

Plots

Confirm that:

☒ The axis labels state the marker and fluorochrome used (e.g. CD4-FITC).

☒ The axis scales are clearly visible. Include numbers along axes only for bottom left plot of group (a 'group' is an analysis of identical markers).

☒ All plots are contour plots with outliers or pseudocolor plots.

☒ A numerical value for number of cells or percentage (with statistics) is provided.

Methodology

Sample preparation	Human CD34+ cells in culture and murine splenocytes
Instrument	BD FACSCaliburTM
Software	FCS Express 6 Flow (De Novo Software)
Cell population abundance	n/a: no sorting was conducted
Gating strategy	For human CD34+ cell cultures, initial gating was on viable fraction based on FSC/SSC characteristics. For murine splenocytes, initial gating was on the Ter119+ fraction. For imaging cytometry, gating was on viable in-focus singlet cells.

☒ Tick this box to confirm that a figure exemplifying the gating strategy is provided in the Supplementary Information.
