# Supplementary material for: Iron control of erythroid microtubule cytoskeleton as a potential target in treatment of iron-restricted anemia
Source: Nat Commun. 2021 Mar 12;12:1645. doi: 10.1038/s41467-021-21938-2 (PMC7955080; doi:10.1038/s41467-021-21938-2)
Supplement: Supplementary file 4 — Source Data [file 41467_2021_21938_MOESM4_ESM.zip › Source Data/Immunoblots Uncropped.pdf]

Main Figures

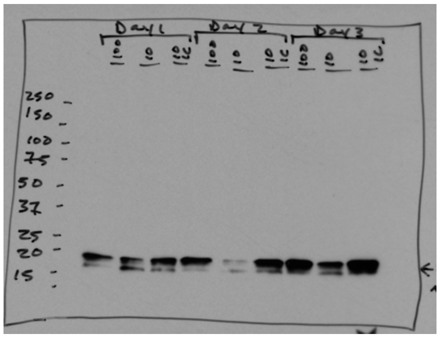

Fig. 3b. Anti-FTH1

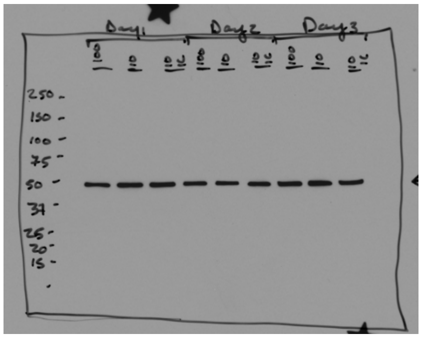

Fig. 3b. Anti-Tubulin

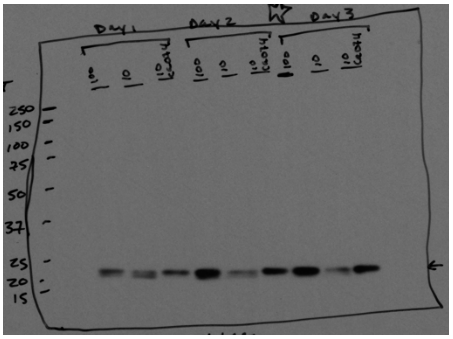

Fig. 3c. Anti-FTH1

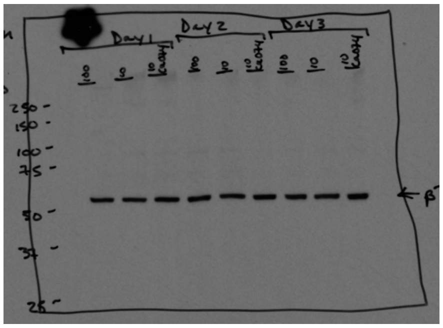

Fig. 3c. Anti-Tubulin

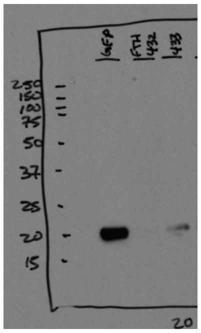

Fig. 3d. Anti-FTH1

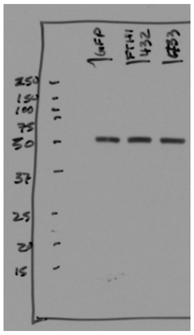

Fig. 3d. Anti-Tubulin

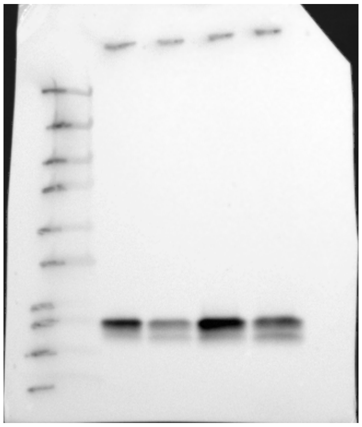

Fig. 4g. Anti-FTH1

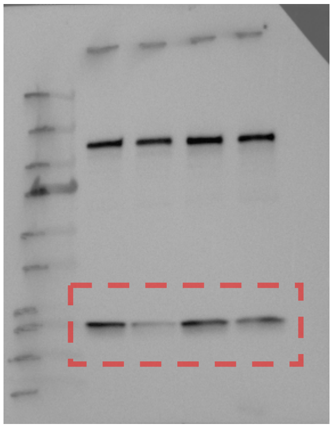

Fig. 4g. Anti-FTL

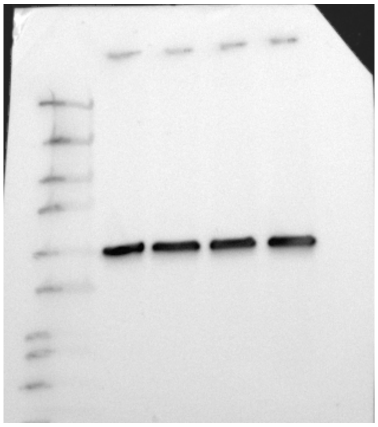

Fig. 4g. Anti-Tubulin

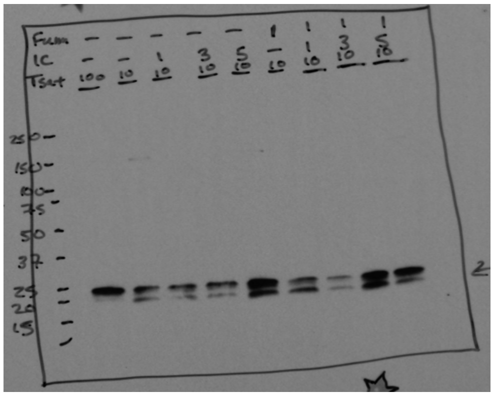

Fig. 5a. Anti-FTH1

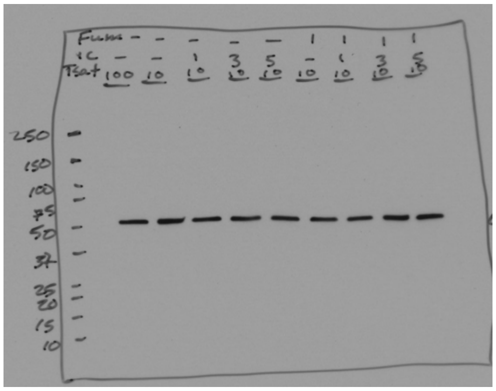

Fig. 5a. Anti-Tubulin

Extended Data Figures

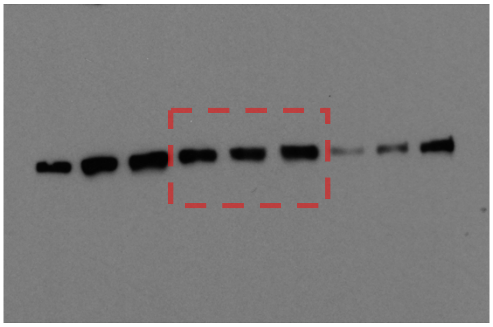

ED Fig. 5a. Anti-AcK40 TUBA

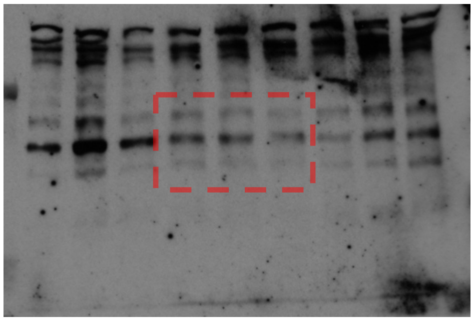

ED Fig. 5a. Anti-deY TUBA

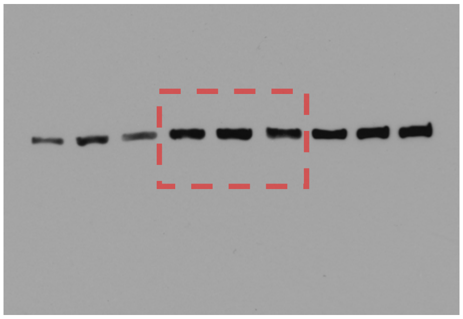

ED Fig. 5a. Anti-TUBA

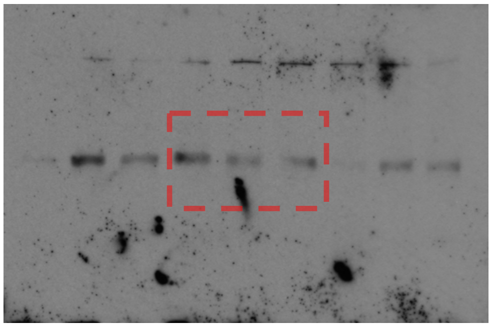

ED Fig. 5a. Anti-AcK379 TUBB

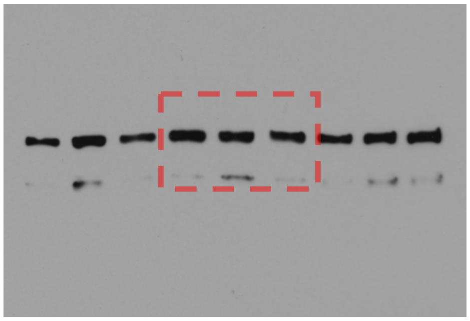

ED Fig. 5a. Anti-TUBB2A

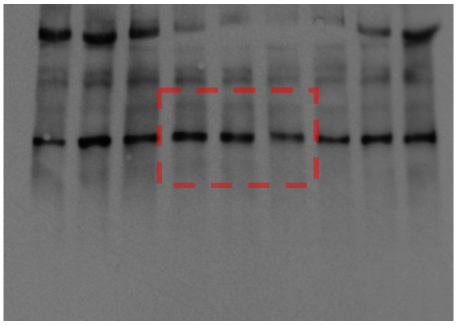

ED Fig. 5a. Anti-TUBB

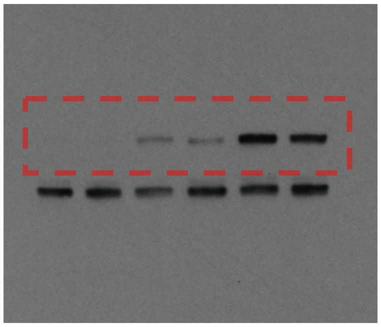

ED Fig. 5b. Anti-TUBB2A

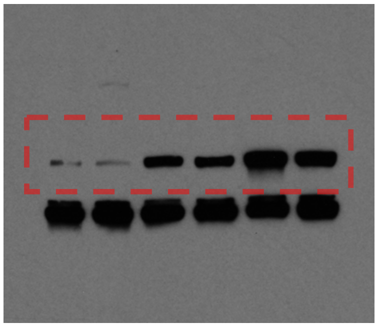

ED Fig. 5b. Anti-TUBB2A (Darker)

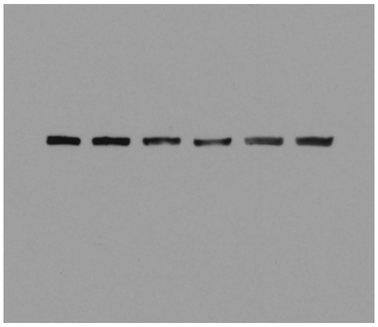

ED Fig. 5b. Anti-TUBA

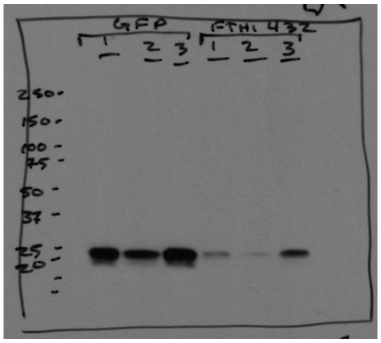

ED Fig. 5c. Anti-FTH1

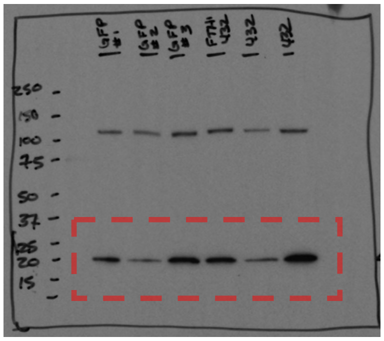

ED Fig. 5c. Anti-FTL

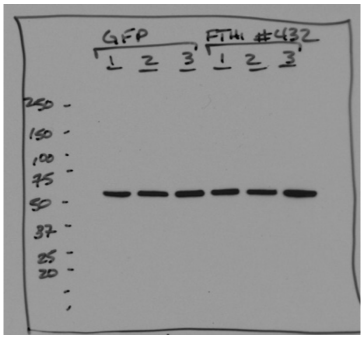

ED Fig. 5c. Anti-TUB

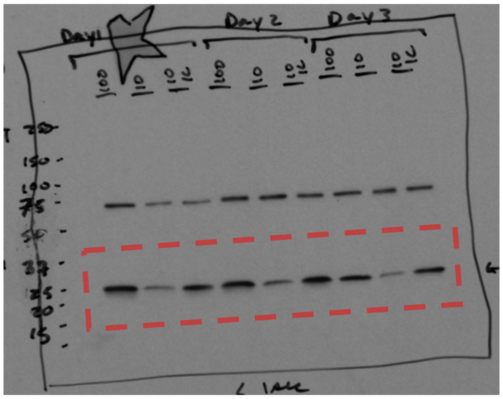

ED Fig. 5d. Anti-FTL

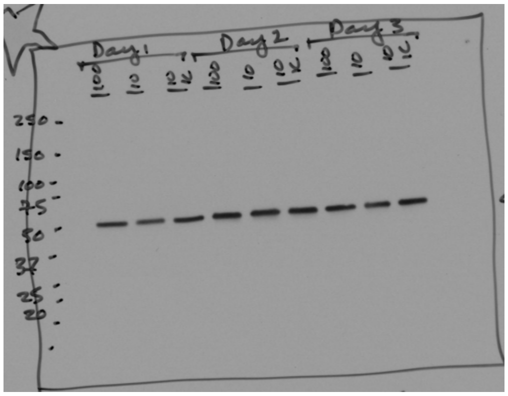

ED Fig. 5d. Anti-TUB

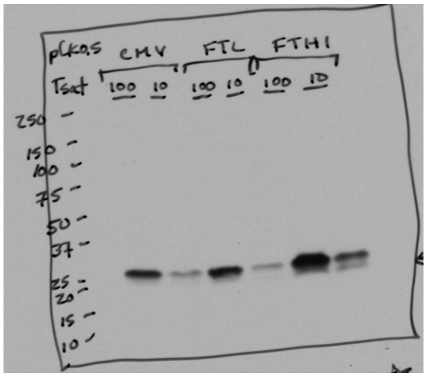

ED Fig. 8b. Anti-FTH1

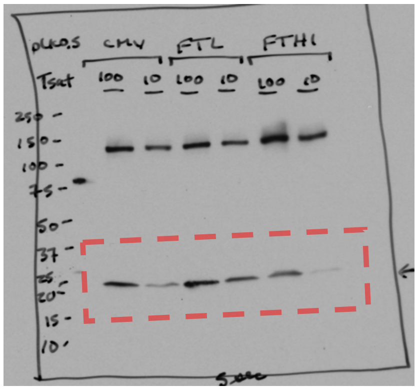

ED Fig. 8b. Anti-FTL

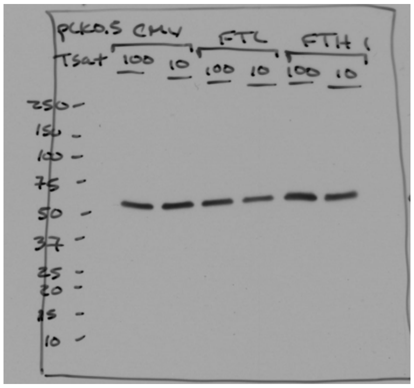

ED Fig. 8b. Anti-TUB

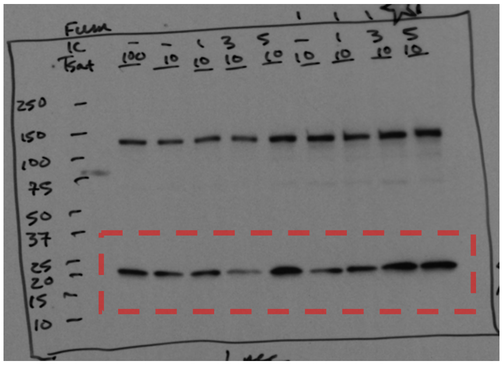

ED Fig. 9a. Anti-FTL

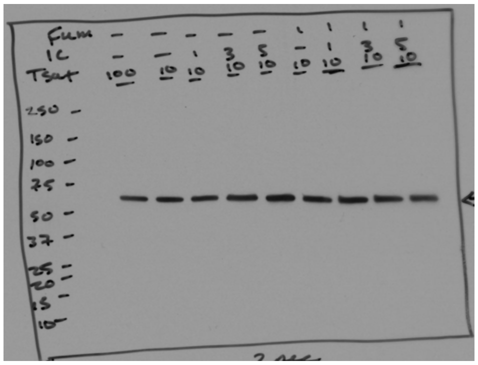

ED Fig. 9a. Anti-TUB

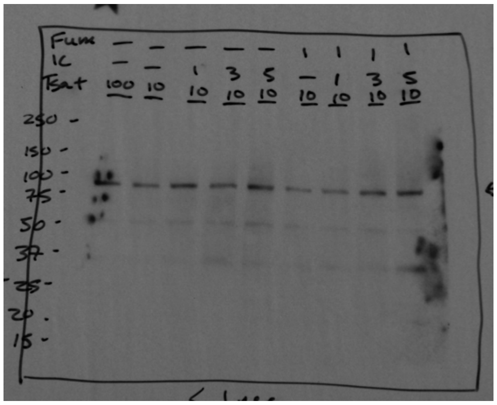

ED Fig. 9e. Anti-Nrf2

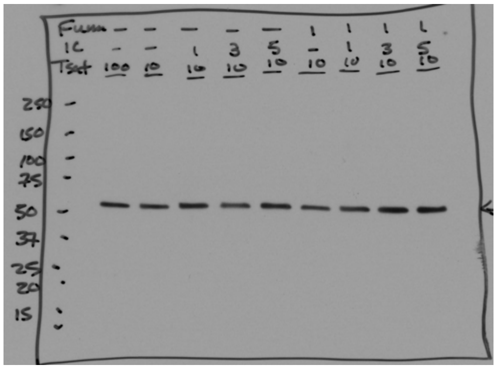

ED Fig. 9e. Anti-TUB
